# “Perspective” – A new approach to serve our Light community

**DOI:** 10.1038/s41377-018-0085-y

**Published:** 2018-11-14

**Authors:** Dayong Jin, Jianlin Cao

**Affiliations:** 1 Faculty of Science, Institute for Biomedical Materials and Devices (IBMD), University of Technology Sydney, Sydney, New South Wales 2007 Australia; 2 State Key Laboratory of Applied Optics, CIOMP, CAS. No. 3888, Dongnanhu Road, Changchun, China

Scientists are known as the idea explorers, technology inventors and visionaries for the great benefits of our end-users and society. They are optimistic, realistic and scientifically rigorous. The way for them to create new ideas and technologies can be through their intuitions and others’ guidance and inspirations. Therefore scientific publishing plays an essential role in effective communications and knowledge advancements. For the past decade, rapid growth occurred in our science communities and the number of research outputs. Merely pursuing quantity of publishing may be in lack of satisfaction, given the large volume of information produced becomes overwhelming to our early career researchers, students or cross-disciplinary researchers. Top-tier journals should stay more closely connected with our most creative authors and influential research leaders, and find ways to boost more interdisciplinary discussions and inspire creative thoughts and practice.

Since its first launch in 2012, *Light: Science and Applications* has achieved remarkable successes, with 434 high-quality research papers and 34 reviews published, attracted a total of more than 14,000 citations to date. To facilitate the rapid growth in multidisciplinary research, in 2017 we launched the column of “News & Views”. To enhance our capacity as the channel to connect leading research labs, and to keep the broad scientific communities informed of emergence of new research directions, we have decided to introduce the current new column Perspective.

While a Review provides a broad and comprehensive overview of a topic and the News and Views article targets general audience for overall knowledge, Perspective is providing timely and insightful thoughts and ideas from the most active peers with new directions of research, opportunities and challenges in emerging fields. Our editorial board will regularly invite research leaders to contribute to this column, and particularly welcomes proposals from the *Light* reviewers and regular authors.

Each paper published in Perspective denotes a scholarly discussion of one major breakthrough arisen recently or a summary of the progress to highlight the emerging opportunities in light sciences, technologies and applications. The papers published in Perspective may reveal opinions unique to individuals or specific areas, but being impartial is our key principle. We encourage articles in the form of joint commentary incorporating multiple labs and/or disciplines, and also intend to empower discussions and considerations of new approaches to investigations and understandings of commonly interested research topics.

A Perspective paper needs to be condensed with a limitation of 2000 words, 20 references and no more than two illustrative display items. The suggested format begins with a leading paragraph of up to 75 words to brief the breakthrough, and contains a mini-review of 300 words to summarize the latest advances, a discussion section listing the relevant areas, scopes and gaps in the field(s) for further investigation/solutions and the trend of the field development; as well as an outlook section to showcase potential applications and implications of the field.

Because of its forward-looking style, roadmap-style illustrations are preferred, where it is essential to perform a systematic analysis and demonstrate thorough understandings of the factors having potential impact on the emerging field. It is also our intention to capture the major ideas arising at the planning stage of a major grant application, or observations and opinions at the operational stage of a multidisciplinary research initiative/centre. In view of this, *Light*: Perspective can stay upfront of the latest progress of the major research programs, facilitate interdisciplinary and international collaborations.

Science is not only about bold findings, it is significantly associated with scientists and proactive engagement. With our first Perspective article recently published (https://www.nature.com/articles/s41377-018-0058-1), we hope the *Light*: Perspective column becomes a forum for our community to illustrate the many future emerging fields!

